# Targeted Delivery of Engineered RVG-BDNF-Exosomes: A Novel Neurobiological Approach for Ameliorating Depression and Regulating Neurogenesis

**DOI:** 10.34133/research.0402

**Published:** 2024-07-04

**Authors:** Shaobo Liu, Lei Chen, Mei Guo, Yongbiao Li, Qingshan Liu, Yong Cheng

**Affiliations:** ^1^ Key Laboratory of Ethnomedicine for Ministry of Education, Center on Translational Neuroscience, Minzu University of China, Beijing 100081, China.; ^2^ College of Life and Environmental Sciences, Minzu University of China, Beijing 100081, China.; ^3^ Institute of National Security, Minzu University of China, Beijing 100081, China.

## Abstract

Addressing the urgent need for innovative depression treatments, this study heralds a breakthrough in major depressive disorder (MDD) therapy by intertwining clinical observations with neurobiological advancements. We analyzed brain-derived neurotrophic factor (BDNF) levels in serum exosomes from a diverse group of 60 individuals, including first-episode, drug-free MDD patients, medicated MDD patients, and healthy controls. Our results revealed a significant decrease in BDNF levels within MDD patients’ exosomes, which notably increased post-medication, highlighting BDNF’s potential as a biomarker for both MDD diagnosis and treatment efficacy. Advancing these clinical findings, we developed RVG-modified exosomes engineered to overexpress BDNF (RVG-BDNF-Exos), designed to directly target neuronal cells. Our findings demonstrate that these engineered exosomes can successfully traverse the blood–brain barrier, targeting neurons in the hippocampus and prefrontal cortex. In our mouse model of depression induced by lipopolysaccharide, RVG-BDNF-Exos treatment led to a significant increase of BDNF in these key brain regions, crucial for mood regulation and neurogenesis. This intervention modulated the BDNF/TrkB/AKT signaling pathway, central to neural plasticity and implicated in depression’s pathogenesis. Behavioral assessments exhibited substantial improvements in depressive-like behaviors in mice treated with RVG-BDNF-Exos, including reduced immobility in Tail Suspension and Forced Swim Tests. Additionally, our treatment effectively decreased neuroinflammation, as evidenced by the reduction in microglia and astrocyte numbers. Moreover, RVG-BDNF-Exos treatment enhanced neurogenesis and regulated synaptic plasticity, as indicated by the increased expression of neuronal markers MAP2 and DCX, and synaptic proteins PSD95 and Syn-1. In conclusion, this study not only underscores the clinical potential of serum exosomal BDNF as a diagnostic and therapeutic marker for MDD but also demonstrates the efficacy of RVG-BDNF-Exos in alleviating depressive symptoms. Our findings pave the way for future targeted, personalized psychiatric treatments, offering a promising direction in MDD therapy.

## Introduction

Major depressive disorder (MDD), a widespread psychiatric ailment, manifests as sustained moodiness. Forecasts from various studies suggest that by 2030, MDD will emerge as a leading cause of mortality and disability globally [1]. Despite extensive research and treatment advances, approximately half of patients with MDD fail to achieve complete remission with long-term antidepressant therapy, which is often accompanied by marked side effects [2,3]. This underscores the critical need for innovative and effective treatment strategies for depression.

Brain-derived neurotrophic factor (BDNF), a neurotrophin initially isolated from brain of the pigs by Barde in the year 1982 [4], is a 27-kDa polypeptide crucial for the development, differentiation, and survival of neurons. The role of BDNF in regulating neural plasticity and its involvement in MDD pathogenesis have been substantiated by numerous studies [5,6]. BDNF is known for its high concentration in the cortex and hippocampus, where it plays a pivotal role in neurological development and mitigating depression via specific pathways [7]. In the postmortem brain samples from MDD patients, reduced precursor BDNF (proBDNF), quantified by immunoautoradiography, was detected in the right hippocampus [8]. BDNF expression was significantly lower in the brains of MDD patients than in those of sex-matched control subjects and antidepressant-untreated MDD subjects [9].

The expression levels of BDNF were significantly reduced in both the serum of patients with MDD and in the brains of animal models exhibiting depressive symptoms [10,11]. Furthermore, studies have shown that serum and exosomal BDNF levels are lower in MDD patients than in healthy individuals [12]. Experimental models, such as intraperitoneal lipopolysaccharide (LPS) injection in rats, mimic depression-like behavior and are associated with a marked reduction in BDNF mRNA and protein expression in the hippocampus [13]. Intriguingly, direct hippocampal BDNF administration in rat models has produced notable antidepressant-like effects [14]. Recent evidence also suggests that intranasal BDNF administration can reduce pro-inflammatory cytokine levels and enhance neurogenesis in mice subjected to chronic unpredictable mild stress (CUMS) [15], highlighting the therapeutic potential of elevating BDNF levels in the brain. However, effective and targeted delivery of BDNF to the brain continues to be a substantial challenge.

Exosomes, which are 30 to 150 nm in size, encapsulate various bioactive components and play a critical role in material transport [16–18]. They have been proposed as a potential delivery system for neural remodeling in depression [13,19,20]. However, the lack of target specificity limits their clinical applicability. Recent advances have seen the development of dual-function targeted nanodrug extracellular vesicles, including siRNA targeting YTHDF1, which exhibit enhanced tumor targeting and endolysosomal escape [21]. Likewise, hepatocyte-derived exosomes have been utilized for liver-targeted CRISPR-Cas9 RNP delivery [22]. The developed cGAMP@dual-anti-Exos employ biocompatible exosomes as targeted drug carriers, facilitating the delivery of a range of immune drugs to the target [23]. However, these approaches have not successfully addressed neuron-specific targeting. To overcome this, recent studies have explored the engineering of exosomes with rabies virus glycoprotein (RVG) through lysosome-associated membrane glycoprotein 2b (Lamp2b-RVG), aiming for neuron-specific delivery [24]. In the present study, we hypothesized that engineered RVG-modified exosomes overexpressing BDNF could effectively target neurons.

## Results

### Reduced BDNF levels in patients with MDD

**Fig. 1.**
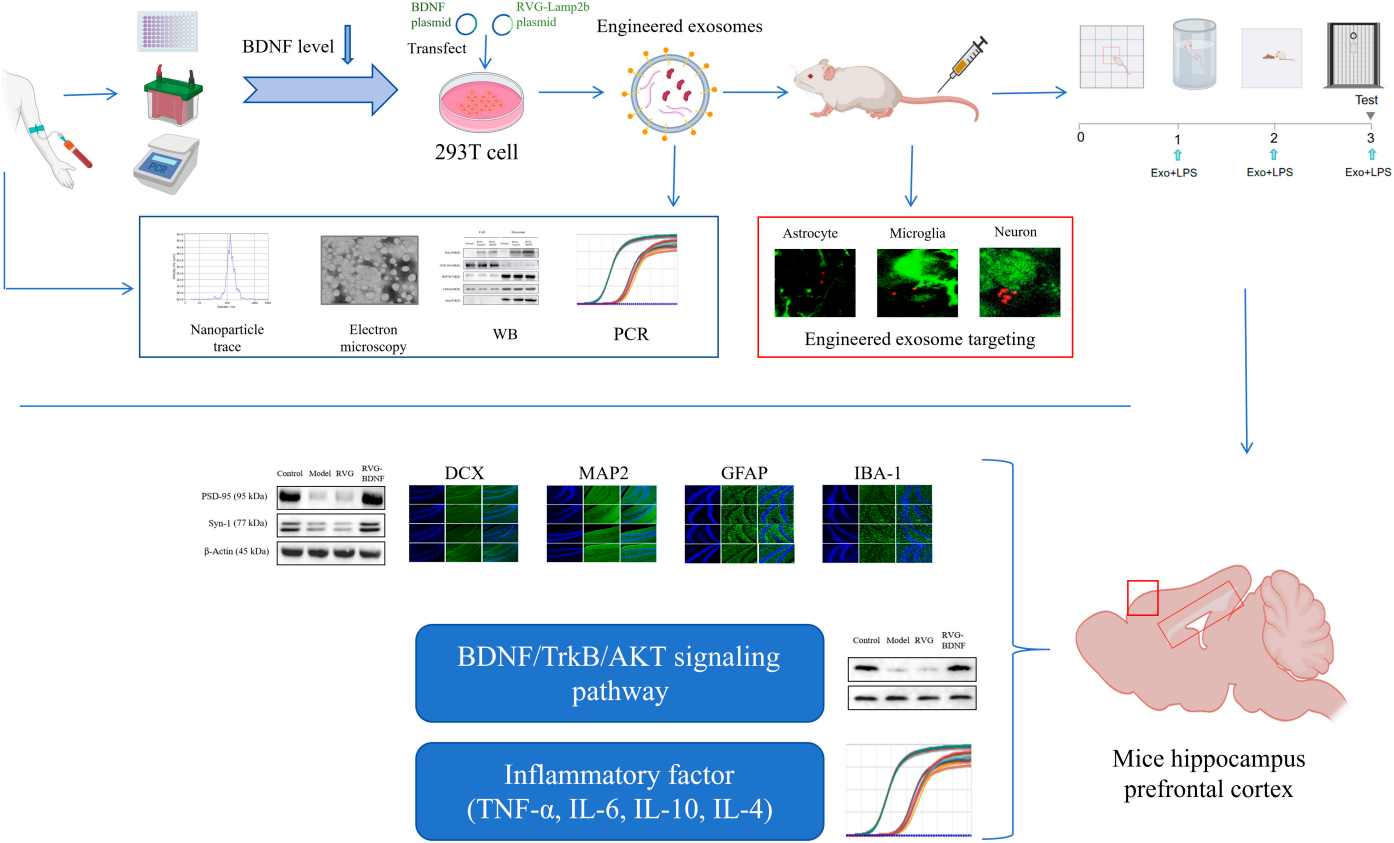
Flowchart of the experimental research program.

**Fig. 2.**
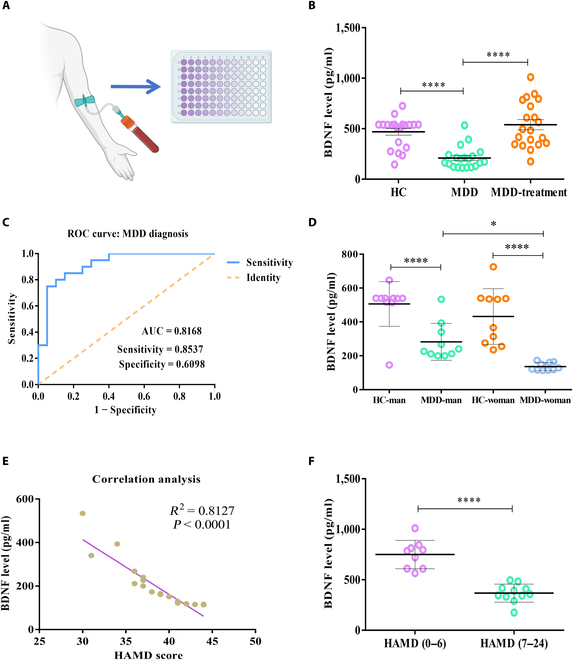
Down-regulation of BDNF in serum exosomes of patients with MDD. (A) Schematic diagram of clinical sample collection and ELISA testing. (B) The BDNF levels in serum exosomes of 20 first-episode, drug-free MDD patients, 20 medicated MDD patients, and 20 healthy control (HC) subjects. *n* = 20 samples/group [*F*_(2, 57)_ = 21.43, *P* < 0.0001]. (C) ROC curve for individual BDNF to separate patients with MDD from HC. (D) Relative serum exosomes BDNF levels in male or female MDD patients and HC [*F*_(3, 36)_ = 18.94, *P* < 0.0001]. (E) Correlation analysis between HAMD scores and BDNF levels in MDD patients revealed a significant negative correlation. (F) Relative serum exosome BDNF levels in medicated patients with good (mRS 0 to 6, *n* = 10) outcomes versus poor (mRS 7 to 24, *n* = 10) outcomes (*P* < 0.0001). Two groups were compared using *t* test, while 3 or more groups were compared using one-way ANOVA followed by Tukey’s multiple comparison test. *P < 0.05, ****P < 0.0001.

A series of experiments were conducted to validate our hypothesis (Fig. 1). We first characterized the extracted human serum exosomes (Fig. S1A to C). In this study, the serum exosome levels of BDNF were measured in a cohort of 60 individuals (Fig. S1D to F and Fig. 2). This group included 20 first-episode, drug-free MDD patients, 20 medicated MDD patients, and 20 healthy control (HC) subjects, as outlined in Fig. 2A and B and Table S1. Notably, Fig. 2B indicates a significant decrease in BDNF content within the serum exosomes of MDD patients. Following treatment, a remarkable increase in BDNF levels within these exosomes was observed, suggesting a therapeutic response. The diagnostic potential of serum exosomal BDNF levels for MDD patients was assessed by receiver operating characteristic curve (ROC) curve analysis. The analysis demonstrated a notable area under the curve (AUC) of 0.8168, with a sensitivity of 0.8537 and a specificity of 0.6098, as shown in Fig. 2C. This finding underscores the potential of BDNF levels in serum exosomes as a biomarker for MDD. Furthermore, the impact of sex on MDD incidence was explored, as depicted in Fig. 2D. The BDNF levels in serum exosomes from male MDD patients were significantly higher than those in serum exosomes from female patients, suggesting a possible increased susceptibility to MDD in women. This observation aligns with existing literature reports [25] and correlates with the HAMD scores, as detailed in Table S1. Additionally, a correlation analysis between HAMD scores and BDNF levels in MDD patients revealed a prominent negative correlation (*P* < 0.001, *R*^2^ = 0.8127), as illustrated in Fig. 2E. Intriguingly, drug-treated MDD patients with lower HAMD scores displayed significantly higher BDNF levels in their serum exosomes compared to those with higher HAMD scores, as shown in Fig. 2F. This suggests that BDNF-enriched targeted exosomes may have therapeutic potential for MDD.

### Characterization of engineered exosomes

**Fig. 3.**
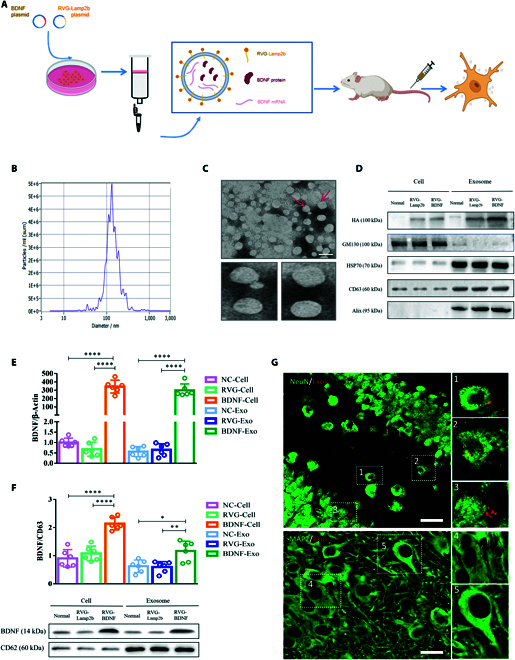
Preparation and characterization of RVG-BDNF-Exos. (A) Scheme diagram of RVG-BDNF-Exos preparation. Plasmid transfection assays were conducted when the 293T cell density reached 70%, and cell supernatants were collected to extract and purify exosomes. (B) Size distribution and concentration of exosomes were analyzed by ZetaView. (C) Transmission electron microscopy images of exosomes. Scale bar, 100 nm. (D) Characteristic expression of engineered exosomes as measured by Western blot analysis. (E) [*F*_(5, 30)_ = 85.36, *P* < 0.0001] and (F) [*F*_(5, 30)_ = 27.08, *P* < 0.0001] Expression level of BDNF was detected in RVG-Vector-Exos or RVG-BDNF-Exos by qRT-PCR (*n* = 6) and Western blot analysis (*n* = 6). The data were normalized to β-actin or CD63 expression and expressed as mean ± SEM. (G) The distribution of RVG-Exos in neurons (NeuN and MAP2) at 8 h after intravenous injection. Three or more groups were compared using one-way ANOVA followed by Tukey’s multiple comparison test. *P < 0.05, **P < 0.01, and ****P < 0.0001. Red dots, Dil + Exos; greenish fluorescent light, NeuN or MAP2, scale bar = 50 μm.

To investigate this hypothesis, we engineered targeted exosomes encapsulated with BDNF. The pcDNA GNSTM-3-RVG-10-Lamp2b-HA plasmid, which contains a peptide derived from RVG fused to Lamp2b, was introduced alongside a BDNF plasmid into 293T cells. This was followed by purification and isolation processes. The targeted delivery of these exosomes was confirmed in mice through tail vein injections, as demonstrated in Fig. 3A. Exosomes were separated from the cell supernatant by qEV columns and verified through nanoparticle tracking analysis, qRT-PCR, and Western blot. The majority of these exosomes were approximately 131 nm in diameter, as determined by nanoparticle tracking analysis, and their structure was confirmed via electron microscopy (EM) (Fig. 3B and C). The presence of the tagged protein HA and exosomal signature proteins including GM130, CD63, HSP70, and Alix was verified, indicating the successful integration of RVG-Lamp2b into the exosomes derived from HEK293T cells (Fig. 3D). The effectiveness of encapsulating BDNF within these exosomes was further analyzed. The qRT-PCR and Western blotting results, shown in Fig. 3E and F, revealed that the RVG-BDNF-Exos group had higher levels of BDNF compared to the RVG-Vector-Exos group, confirming the successful integration of overexpressed BDNF into the exosomes. Finally, to verify the targeting efficacy of the engineered exosomes in the mouse brain, we labeled the exosomes with Dil and administered them via tail vein injection (1×10^10^ particles). The mouse brains were collected, and immunofluorescence staining was conducted 8 h or 3 days post-injection. Figure 3G and Figs. S2 to 5 show that the red Dil particles predominantly localized to the cell membranes of neurons (MAP2 or NeuN), confirming targeted delivery to the desired cells [26].

### RVG-BDNF-exos facilitate BDNF/TrkB signaling in the hippocampus and prefrontal cortex

**Fig. 4.**
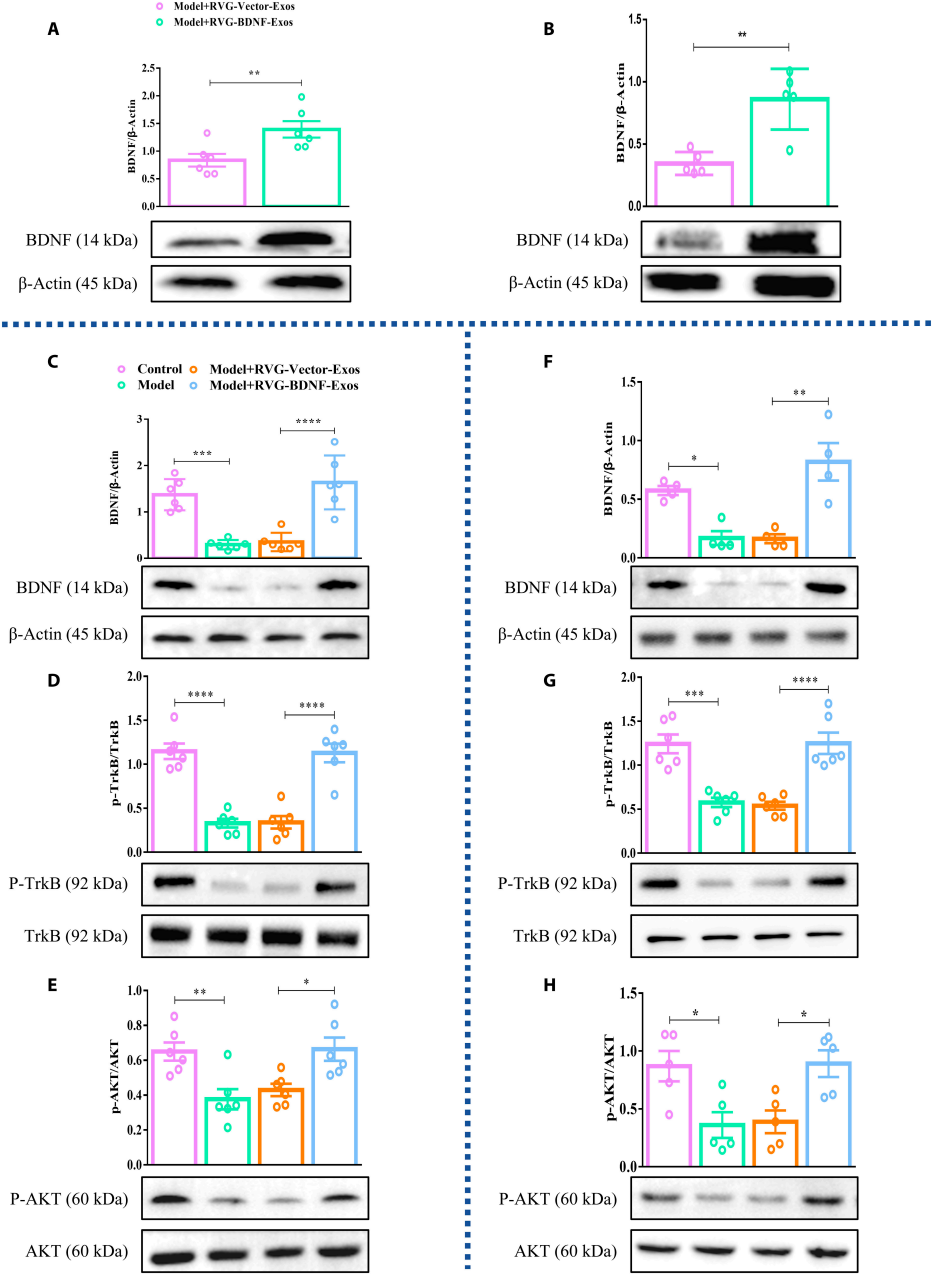
RVG-BDNF-Exos act on BDNF/TrkB signaling pathway in hippocampus and prefrontal cortex. (A and B) Western blot examined the expression levels of BDNF in the hippocampus (A) and prefrontal cortex (B) 3 days after continuous injection of RVG-BDNF-Exos in the tail vein. Western blot experiments tested the effect of RVG-BDNF-Exos for the level of TrkB and Akt proteins of downstream pathways of BDNF (C) [*F*_(3, 20)_ = 22.82, *P* < 0.0001], (D) [*F*_(3, 20)_ = 32.66, *P* < 0.0001] and (E) [*F*_(3, 20)_ = 7.496, *P* = 0.0015] in the hippocampus and prefrontal cortex (F) [*F*_(3, 12)_ = 12.93, *P* = 0.0005], (G) [*F*_(3, 20)_ = 20.38, *P* < 0.0001], and (H) [*F*_(3, 16)_ = 6.521, *P* = 0.0043]. *n* = 4 to 6. Two groups were compared using *t* test, while 3 or more groups were compared using one-way ANOVA followed by Tukey’s multiple comparison test. *P < 0.05, **P < 0.01, ***P < 0.001, and ****P < 0.0001 in comparison with the model or the RVG-Vector-Exos group.

We explored the efficacy of RVG-BDNF-Exos in delivering BDNF to the brain, using a depression model as described previously [27]. RVG-BDNF-Exos or RVG-Vector-Exos were administered intravenously prior to the each LPS injection. Subsequently, Mice received intraperitoneal injections of LPS (1 mg/kg) or an equivalent dose of saline daily for 3 days. Western blot analysis, conducted 3 days post-treatment, revealed a marked increase in BDNF protein levels in both the hippocampus and prefrontal cortex, surpassing those observed in the model group and RVG-Vector-Exos-treated mice (Fig. 4A and B). Binding of BDNF to the TrkB receptor initiates auto-phosphorylation, triggering downstream signaling pathways, including those reducing inflammatory reflects [28]. We assessed the protein expression levels of key components in the BDNF-TrkB pathway. Our data indicated a substantial increase in BDNF expression and enhanced ratios of phosphorylated TrkB (p-TrkB) to TrkB, as well as phosphorylated AKT (p-AKT) to AKT in both the hippocampus and cortex of mice afflicted with depression after treatment with RVG-BDNF-Exos (Fig. 4C to H). The results of these experiments shown that the antidepressant effects of RVG-BDNF-Exos are likely due to regulation of the BDNF/TrkB/AKT signaling pathway.

### Behavioral improvements in mice with depression-like behaviors following RVG-BDNF-exos delivery

**Fig. 5.**
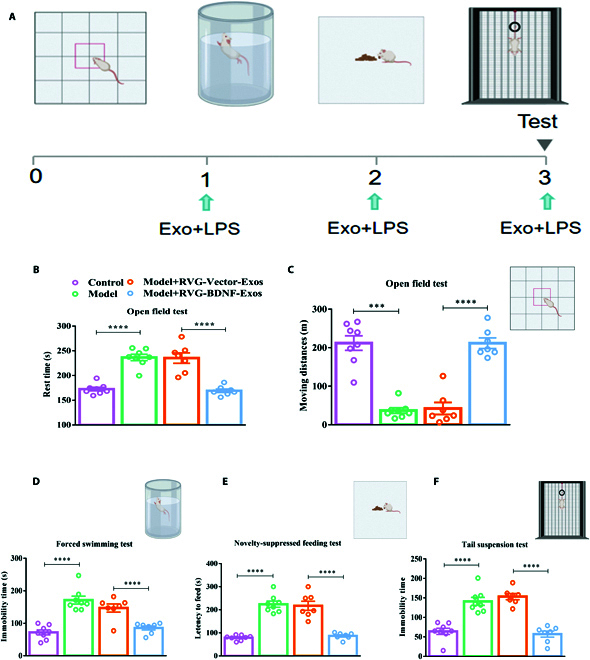
RVG-BDNF-Exos improved depression-like behavior in mice. (A) Schematic of RVG-BDNF-Exos administration and behavioral studies. (B to F) BDNF improved depression-like behavior in depressive mice as measured by the Open Field Test (B and C) [B: *F*_(3, 6)_ = 33.18, *P* < 0.0001; C: *F*_(3, 26)_ = 47.29, *P* < 0.0001], Forced-Swimming Test (D) [*F*_(3, 26)_ = 23.25, *P* < 0.0001], Novelty-Suppressed Feeding Test (E) [*F*_(3, 26)_ = 44.09, *P* < 0.0001], and Tail Suspension Test (F) [*F*_(3, 26)_ = 33.41, *P* < 0.0001]. *n* = 7 to 8 animals/group. Three or more groups were compared using one-way ANOVA followed by Tukey’s multiple comparison test. ***P < 0.001, ****P < 0.0001 versus the model or the RVG-Vector-Exos group.

Behavioral assessments were carried out on the final day of the experiment to evaluate the impact of RVG-BDNF-Exos on depressive-like behaviors in mice (Fig. 5A). Mice receiving RVG-BDNF-Exos treatment exhibited a significant decrease in immobility during the Open Field Test compared to both the RVG-Vector-Exos group and the model group (Fig. 5B and C). Furthermore, in the Forced Swimming Test, the immobility duration was notably reduced in the RVG-BDNF-Exos group (Fig. 5D). During the Novelty-Suppressed Feeding Test, following a 24-h fasting period, the mice treated with RVG-BDNF-Exos demonstrated a reduced latency to feed in a novel environment (Fig. 5E). Additionally, the Tail Suspension Test results (Fig. 5F) indicated prolonged immobility in the RVG-Vector-Exos and model groups, which was effectively alleviated by RVG-BDNF-Exos treatment. Meanwhile, in the chronic restraint stress (CRS) model, we observe similar results (Fig. S6). Collectively, these observations indicate that administering RVG-BDNF-Exos leads to a notable improvement in depressive-like behavior.

### RVG-BDNF-exos mitigate microglial numbers and neuroinflammation in mice with depression-like behaviors

**Fig. 6.**
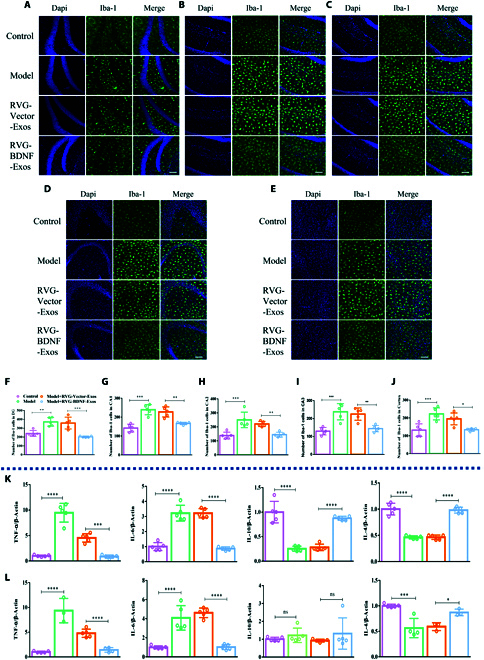
RVG-BDNF-Exos reduced the number of microglia and neuroinflammation in the brain of mice with depression-like behaviors. (A to J) Immunofluorescence staining microglia and cell quantification in DG (A and F) [*F*_(3, 16)_ = 17.72, *P* < 0.0001], CA1 (B and G) [*F*_(3, 16)_ = 23.43, *P* < 0.0001], CA2 (C and H) [*F*_(3, 16)_ = 14.41, *P* < 0.0001], CA3 (D and I) [*F*_(3, 16)_ = 15.40, *P* < 0.0001], and prefrontal cortex (E and J) [*F*_(3, 16)_ = 11.58, *P* = 0.0003] of mice in each group. The qRT-PCR experiments examined the effects of RVG-BDNF-Exos on inflammatory factors (TNF-α, IL-6, IL-10, and IL-4) in the hippocampus (K) [TNF-α: *F*_(3, 16)_ = 78.00, *P* < 0.0001; IL-6: *F*_(3, 16)_ = 78.17, *P* < 0.0001; IL-10: *F*_(3, 16)_ = 53.77, *P* < 0.0001; IL-4: *F*_(3, 16)_ = 101.9, *P* < 0.0001] and prefrontal cortex (L) [TNF-α: *F*_(3, 16)_ = 43.96, *P* < 0.0001; IL-6: *F*_(3, 16)_ = 37.74, *P* < 0.0001; IL-10: *F*_(3, 16)_ = 0.7164, *P* < 0.0001; IL-4: *F*_(3, 11)_ = 15.54, *P* = 0.0003]. *n* = 5. Three or more groups were compared using one-way ANOVA followed by Tukey’s multiple comparison test. *P < 0.05, **P < 0.01, ***P < 0.001, and **** *P* < 0.0001 in comparison with the model or the RVG-Vector-Exos group, scale bar = 100 μm.

Recent studies have highlighted the important role of inflammation in activating microglia (microgliosis) in the brain [29]. In light of this, we focused on quantifying microglia in specific brain regions of mice with depression-like behaviors. We investigated the effect of BDNF on the microglial population in the hippocampal and prefrontal cortical areas of our model mice. On the third day after RVG-BDNF-Exos administration, a significant reduction in microglia was observed in the DG region (Fig. 6A and F). This reduction was also evident in the CA1, CA2, and CA3 regions of mice treated with RVG-BDNF-Exos (Fig. 6B to D and G to I), as well as in the prefrontal cortex (Fig. 6E and J). Also, we observed that RVG-BDNF-Exos significantly attenuated microglia activation (Fig. S7). Moreover, we evaluated pro-inflammatory and anti-inflammatory cytokine levels in these brain regions. After RVG-BDNF-Exos treatment, a reduction in pro-inflammatory cytokines and an elevation in anti-inflammatory cytokines were observed, suggesting an immunosuppressive effect (Fig. 6K and L). In addition, we observed similar results in blood inflammatory factors and spleen (Fig. S8). These results may be related to the fact that RVG-BDNF-Exos can be taken up by microglia in small portions (Fig. S9A). Overall, these results indicate that RVG-BDNF-Exos effectively reverses microglia proliferation and mitigates neuroinflammation.

### RVG-BDNF-exos reduce astrocyte numbers in mice with depression-like behaviors

**Fig. 7.**
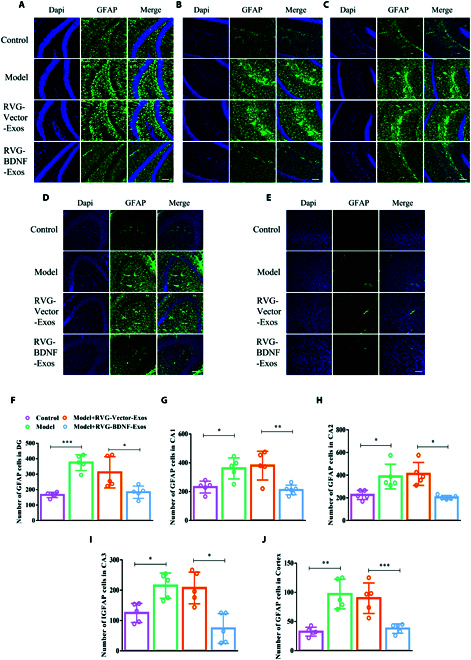
RVG-BDNF-Exos reduced the number of astrocyte in the brain of mice with depression-like behaviors. (A to J) Immunofluorescence staining astrocyte and cell quantification in DG (A and F) [one-way ANOVA, *F*_(3, 16)_ = 13.87, *P* = 0.0001], CA1 (B and G) [*F*_(3, 17)_ = 9.295, *P* = 0.0007], CA2 (C and H) [*F*_(3, 17)_ = 9.597, *P* = 0.0007], CA3 (D and I) [*F*_(3, 16)_ = 11.70, *P* = 0.0003], and prefrontal cortex (E and J) [*F*_(3, 16)_ = 15.73, *P* < 0.0001] of mice in each group. *n* = 5. Three or more groups were compared using one-way ANOVA followed by Tukey’s multiple comparison test. *P < 0.05, **P < 0.01, and ***P < 0.001 in comparison with the model or the RVG-Vector-Exos group, scale bar = 100 μm.

Astrocytes, implicated in the progression of depression due to their dysfunction [30], are characterized by numerous protrusions that interact with nerve cell membranes. We further explored the influence of RVG-BDNF-Exos on astrocytes. Initially, we assessed the expression of GFAP, an astrocyte marker, in the DG areas of mice across all experimental groups. In both the model and RVG-Vector-Exos groups, a notable increase in GFAP-positive cells was visualized around the DG. However, following treatment with RVG-BDNF-Exos, the number of GFAP-positive cells in the DG region approached levels comparable to those in the control group (Fig. 7A and F). A similar trend in GFAP expression was observed in the CA1, CA2, and CA3 regions (Fig. 7B and D and G to I) and in the prefrontal cortex (Fig. 7E and J). These results may be related to the fact that RVG-BDNF-Exos can be taken up by astrocytes in small portions (Fig. S9B). These findings indicate that RVG-BDNF-Exos treatment reduces astrocyte numbers.

### RVG-BDNF-exos enhance neurogenesis and regulate synaptic plasticity, contributing to depression amelioration

**Fig. 8.**
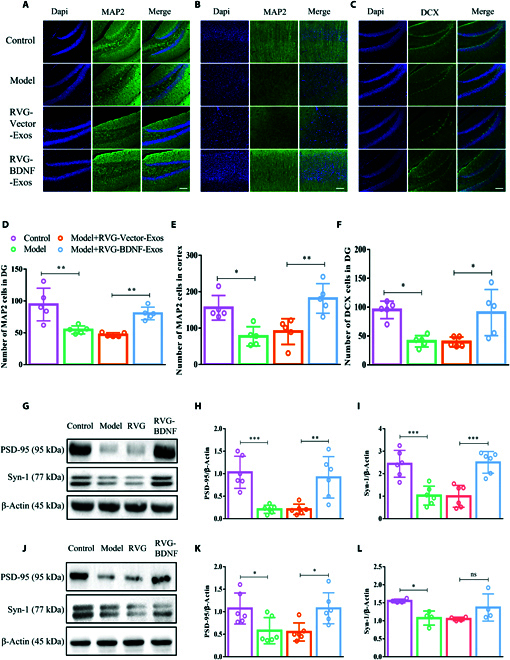
RVG-BDNF-Exos promoted neuronal regeneration. (A to J) Immunofluorescence staining MAP2 and DCX with cell quantification in DG (A, D and C, F), and prefrontal cortex (B and E) of mice in each group [D: *F*_(3, 16)_ = 12.20, *P* = 0.0002; E: *F*_(3, 16)_ = 10.63, *P* = 0.0004; F: *F*_(3, 16)_ = 9.286, *P* = 0.0009]. (G to L) Western blot examined the expression levels of synapse-associated protein PSD-95 and Syn-1 in the hippocampus (G to I) [H: *F*_(3, 20)_ = 13.18, *P* < 0.0001; I: *F*_(3, 20)_ = 17.07, *P* = 0.0004] and prefrontal cortex (J to L) [K: *F*_(3, 20)_ = 5.743, *P* = 0.0053; L: *F*_(3, 12)_ = 5.002, *P* = 0.0177] 3 days after continuous injection of RVG-BDNF-Exos in the tail vein. *n* = 4 to 6. Three or more groups were compared using one-way ANOVA followed by Tukey’s multiple comparison test. *P < 0.05, **P < 0.01, and *** *P* < 0.001 in comparison with the model or the RVG-Vector-Exos group, scale bar =100 μm.

Neurons, recognized as the fundamental units of the nervous system, are key in countering depressive and anxiety-like behaviors [31]. In our study, we used MAP2, a marker for mature neurons, and DCX, a microtubule-associated phosphoprotein crucial for neurogenesis, to assess neuronal development. Our findings revealed a substantial increase in MAP2-positive neurons in the DG and prefrontal cortex of mice treated with RVG-BDNF-Exos, in contrast to the model and RVG-Vector-Exos groups (Fig. 8A, B, D, and E). Correspondingly, DCX expression mirrored MAP2 results; the RVG-BDNF-Exos group showed a remarkable increase in DCX-positive cells in the DG (Fig. 8C and F), indicating enhanced neurogenesis.

Intrigued by these results, we further explored the potential impact of RVG-BDNF-Exos on synaptic plasticity. We analyzed the expression levels of synapse-associated proteins, involving PSD95 and Syn-1, in both the hippocampus (Fig. 8G to I) and prefrontal cortex (Fig. 8J to L). Post-treatment with RVG-BDNF-Exos, significant changes in synaptic protein expression were detected in the hippocampal region. Notably, in the prefrontal cortex, PSD95 showed significant alterations. These results strongly indicate that RVG-BDNF-Exos may enhance depression treatment efficacy by promoting neurogenesis and regulating synaptic plasticity.

## Discussion

In this groundbreaking study, we successfully developed engineered exosomes that overexpress BDNF, showing a specific affinity for neuronal cells. This innovative approach represents a substantial advancement in neurotherapeutics, enabling the efficient delivery of BDNF directly to neurons within the hippocampus and prefrontal cortex, areas critically affected in depression. The introduction of these neuron-targeted exosomes through tail-vein injection represents a pivotal development in treating neurological disorders, particularly depression.

The remarkable ability of RVG-BDNF-Exos to alleviate depression-like symptoms in mice, induced by intraperitoneal LPS injection, is a testament to the therapeutic potential of BDNF in mood regulation and neurogenesis. This finding is particularly important, because it addresses the crucial gap in current treatment methods for depression. Our approach ensures the targeted delivery of BDNF to neurons, providing an innovative solution to the challenges often faced in traditional systemic drug delivery. Furthermore, altered expression levels of BDNF, TrkB, and synaptotagmin 1 genes in exosomes derived from the hippocampus, prefrontal cortex, and serum of depressed rats underscore their significance in MDD [32].

Exosomes, as vital mediators of intercellular communication, transport various genetic materials, including proteins, mRNAs, and miRNAs. Their role in the pathological processes associated with depression is increasingly recognized. For instance, microglia-enriched miR-143a-5p and MDD patient-derived miR-139-5p, known for inhibiting neurogenesis in mice with depression-like behaviors, highlight the potential of exosomes in MDD pathophysiology [19,20]. The advancements in targeted drug delivery, such as engineered extracellular vesicles (EVs), are a critical area of focus in modern medicine. Our research aligns with these developments, demonstrating the potential of engineered exosomes in targeting neuronal cells, thus opening up new possibilities for treatments in neurology and beyond. Recent studies have constructed EVs with precise targeting specificity for various cell types, such as endothelial cells, dendritic cells [33], and chondrocytes [34]. Notably, chondrocyte-targeting exosomes (CAP-Exo) have been developed for arthritic rats [34], and bone marrow mesenchymal stem cells (BMSCs) have been engineered for targeted delivery in hypoxic tumor environments [35]. Our study contributes to this body of research by showcasing the potential of engineered exosomes in targeted neuronal delivery, paving the way for innovative treatments in neurology and other medical fields.

Emerging research underscores the presence of neural stem cells within the central nervous system and the continuous occurrence of neurogenesis throughout an individual’s lifespan. This process, crucial for generating new neurons, is closely connected to the pathophysiology of depression and responses to antidepressant treatments [36,37]. Our study’s findings extend beyond mitigating depressive symptoms to significantly promoting neurogenesis via the BDNF/TrkB signaling pathway. This process is crucial for the repair and rejuvenation of brain regions affected by depression. Microglia play a crucial role as immune cells in the central nervous system (CNS) and are closely associated with the pathogenesis and treatment of depression [38]. We observed a notable reduction in the number of microglia and astrocytes, indicating a decrease in neuroinflammation, a known contributor to the pathogenesis of depression. This dual effect of enhancing neurogenesis and reducing neuroinflammation offers a comprehensive approach to treating depression. The positive therapeutic impact of immune cell-derived exosomes on depression, especially in modulating inflammatory responses within the brain, provides a valuable context for our findings. The BDNF/TrkB signaling pathway’s role in neurogenesis and neuroinflammation is well-documented in the literature. Studies, including those on compounds like Ginsenoside Rb1, have shown that the BDNF/TrkB pathway can reverse depression-like behaviors induced by CUMS [39]. Our study adds to this knowledge by elucidating the potential of exogenous BDNF in facilitating neuron outgrowth and regulating synapse formation, emphasizing its critical role in the central nervous system. Recent studies have indicated that the neuron classes determined the microglia density and molecular state acquisition [40]. The findings in the study reveal that these engineered exosomes proficiently delivered BDNF to neurons, alleviating depression-like behavior induced by LPS and reducing neuroinflammation by modulating the BDNF-TrkB signaling pathway.

While our results are promising, our experiments have some limitations. Firstly, behavioral tests conducted at a single time point may not fully capture the long-term effects of RVG-BDNF-Exos treatment on depressive-like behaviors. Secondly, while the study explores the BDNF/TrkB/AKT signaling pathway and its involvement in depression, the exact molecular mechanisms underlying the therapeutic effects of RVG-BDNF-Exos remain to be fully elucidated. The ethical landscape demands stringent adherence to patient safety and informed consent, ensuring that participants are fully aware of the therapy’s risks and benefits and consent to their involvement in clinical trials. In addition, from a regulatory standpoint, navigating through ethical review boards and achieving regulatory approvals are critical steps that require meticulous attention to ensure compliance with ethical, legal, and quality standards, including Good Manufacturing Practice for pharmaceuticals. This ensures the therapy’s quality, safety, and efficacy. Moreover, we acknowledge several potential hurdles in this transition, such as validating the safety and effectiveness of the therapy to preempt adverse reactions and treatment failures. The financial aspects, including production and application costs, pose substantial challenges that need to be addressed to ensure the therapy’s affordability and accessibility. By outlining these considerations, we aim to provide a realistic overview of RVG-BDNF-Exos therapy’s journey toward clinical utilization, highlighting the importance of ethical, regulatory, and logistical planning in bringing innovative treatments to patients.

In conclusion, our study represents a substantial breakthrough in treating depression. The administration of RVG-BDNF-Exos post-depression onset not only offers a novel therapeutic approach but also provides valuable insights into the mechanisms of neurogenesis and neuroinflammation in depression. These findings hold considerable potential for clinical applications, underscoring the importance of continued research in targeted neurotherapeutics. Our work contributes a vital piece to the complex puzzle of neurological disorder treatment, paving the way for groundbreaking research and therapy that could noticeably impact patient care in neurology.

## Methods

### Subjects and sample collection

We enrolled 20 drug-free patients with first-episode MDD patients and 20 patients with MDD after treatment at the Third People’s Hospital of Foshan. Diagnostic interviews were conducted following the criteria outlined in the Diagnostic and Statistical Manual of Mental Disorders, Fifth Edition. Disease severity in MDD patients was scored using the 17-item Hamilton Depression Rating Scale (HAMD). Additionally, 20 HC subjects, who are verified to be free of psychiatric illnesses by trained psychiatrists through advertisements at the Third People’s Hospital of Foshan, were included. Table S1 presents the demographic and clinical characteristics of both patients and HC subjects. All participants provided written informed consent before being included in the study. The research protocol received approval from the Ethics Committee at The Minzu University of China, Beijing, China, and adhered to the principles outlined in the Declaration of Helsinki. Peripheral blood was collected from the participants in the morning, between 7 and 8 AM, using anticoagulation tubes. Serum samples were obtained by centrifugation at 5,000 × *g* for 15 min and preserved at −80°C until further analysis. Exosomes were purified and subjected to a BDNF assay of PCR, Western blot analysis (WB), and a commercially available ELISA kit (SEKH-0101, Beijing Solarbio Science & Technology Company), following the manufacturer,s operating instructions. Serum exosomes were also measured via a protein concentration unit Vivaspin (Sartorius) and EM.

### HEK 293T cell culture

HEK 293T cells were maintained in high-glucose Dulbecco’s modified Eagle’s medium (DMEM, HyClone) supplemented with 10% fetal bovine serum (FBS, Gibco), and antibiotics. The cells were maintained at 37°C with 5% CO_2_. Subsequent plasmid transfection was performed when the cell density reached 70%.

### Primary cultures

Neurons, astrocytes, and microglia were cultured from the hippocampus of 24-h-old mice, following previously described methods [41,42]. Subsequently, these cells were seeded onto 24-well plates (10 × 10^5^ cells/well) with coverslips. Adherent cells were then incubated for 10 days (neurons), 7 days (astrocytes), and 7 days (microglia) before being utilized for the experiments.

### Expression plasmid and transfection

The pcDNA GNSTM-3-RVG-10-Lamp2b-HA plasmid (Addgene) contains the GNSTM glycosylation motif, a 3-residue spacer, an RVG peptide, a 10-residue spacer, Lamp2b (an exosomal transmembrane protein), another 3-residue spacer, and the hemagglutinin (HA) tag. The N-terminal of GNSTM glycosylation motif, RVG peptide targeting acetylcholine receptor, Lamp2 signal peptide, and the C-terminal of HA were inserted into the plasmid. For the BDNF plasmid (Addgene), pcDNA3.1-BDNF-3xFlag, the cloning vector was pcDNA3.1(+) with the BamHI–XhoI cloning digest site. To generate RVG-exosomes, we transfected the pcDNA GNSTM-3-RVG-10-Lamp2b-HA plasmid into 293T cells. Similarly, to produce RVG-BDNF-exosomes, we co-transfected 2 distinct plasmids, namely, the pcDNA GNSTM-3-RVG-10-Lamp2b-HA plasmid and the pcDNA3.1-BDNF-3xFlag plasmid, into 293T cells simultaneously. This ensured the incorporation of both RVG and BDNF into the exosomes secreted by the cells. Subsequently, we conducted PCR, Western blotting, and immunofluorescence analyses to confirm the characterization of the exosomes, thus validating the successful transfection. The specific steps for plasmid transfection are as follows: As previously described [43], to produce exosomes containing RVG or BDNF, we transfected 293T cells using Lipo293 Transfection Reagent (Beyotime) and plasmids containing RVG or BDNF for 30 h to induce overexpression of RVG and/or BDNF in cells. Subsequently, exosomes were collected, which also contained RVG and/or BDNF. The cells were then washed with phosphate buffered saline (PBS) and cultured in medium containing 10% dialyzed FBS (removing exosomes from FBS) for an additional 48 h.

### Purification and characterization of engineered exosomes

As outlined in previous protocols [20,44,45], exosomes were harvested from the cell supernatant or human serum using a qEV column (Izon) and concentrated at 11,000 rpm for 30 min with a protein concentration unit Vivaspin. The quantification of exosomes was performed using the nanoparticle tracer instrument ZetaView (Particle Metrix). For EM, exosomes were redissolved in PBS and visualized via transmission EM. The successful construction of engineered exosomes was confirmed through PCR or WB for qualitative analysis as well.

### Engineered exosome targeting

Consistent with our previous methodology [43], we labeled exosomes with 1,1′-dioctadecyl-3,3,3′,3′-tetramethylindocarbocyanine perchlorate (DiI) (Beyotime) solution and administered the exosomes (1×10^10^ particles) via the tail vein to normal or LPS-induced mice. Eight hours or 3 days later, the mice were sacrificed and the brain slices were examined by immunofluorescence. For primary cells, immunofluorescence experiments were conducted after incubating with Dil-labeled exosomes for 12 h.

### Animals and treatments

Male BALB/c mice (8 to 10 weeks, 22 to 25 g) were procured from Vital River Laboratory (Beijing, China). All animal protocols were ethically confirmed by the Animal Care and Use Committee of Minzu University of China, and were performed in strict accordance with the National Institutes of Health Laboratory Animal Care and Use Guidelines (NIH Publication No. 80-23). Following a protocol based on a prior study with minor adjustments [27], each mouse group (*n* = 7 to 8) received intraperitoneal injections of 0.9% saline or LPS (1 mg/kg) dissolved in sterile 0.9% saline for 3 consecutive days. In the LPS-treated group, the mice were administered injections of saline or exosomes (1×10^10^ particles/each) containing BDNF or RVG, through the tail vein. After 1 h, this treatment was immediately followed by the administration of LPS. Three days later, behavioral tests were conducted on the mice, followed by euthanasia and the collection of the mouse hippocampus, prefrontal cortex, serum, and spleen. The harvested spleens were weighed after they were soaked were soaked in physiological saline. Based on prior research [46], mice subjected to CRS were confined in 50-ml restraint cylinders tailored to their body size, allowing for free breathing, for 21 consecutive days (4 h per day). Throughout this 21-day period, CRS mice received bi-weekly injections via the tail vein, either of saline or exosomes containing RVG or BDNF (at a dosage of 1 × 10^10^ particles each). Correspondingly, control mice received bi-weekly saline injections via the tail vein. After the 21-day duration, all experimental mice underwent behavioral assays to evaluate the effects of RVG-BDNF-Exo.

### Behavioral tests

After LPS, CRS, or engineered exosome administration, we conducted the open field, forced swim, and tail suspension tests, following established protocols [20,47,48]. For the novelty suppression of feeding test, we assessed the latency of the animals to approach and consume familiar food in a novel environment. Prior to testing, the mice were fasted for 24 h. Food pellets were positioned in the center of the test field, and the mice were introduced into a corner of the field and allowed to explore for up to 5 min. The time taken for the mice to initiate exploration and begin chewing the food was meticulously recorded.

### WB analysis

Total proteins were obtained from engineered exosomes, mouse hippocampus, and prefrontal cortex using the RIPA lysis buffer (Beyotime), following established protocols [49,50]. The total proteins were separated by SDS–polyacrylamide gel electrophoresis and run on 8%–15% gels. The primary antibodies used for incubation were rabbit anti-β-Actin (1:1,000, Cell Signaling), mouse anti-Postsynaptic Density Protein 95 (PSD95) (1:1,000, Sigma), rabbit anti-Synapsin-1 (Syn-1) (1:1,000, Abcam), mouse anti-BDNF (1:1,000, Cell Signaling), rabbit anti-TrkB (1:1,000, Cell Signaling), rabbit anti-p-TrkB (1:1,000, Cell Signaling), rabbit anti-Akt (1:1,000, Abcam), rabbit anti-p-AKT (1:1,000, Abcam), rabbit anti-GM130 (1:1,000, Cell Signaling), mouse anti-CD63 (1:1,000, Cell Signaling), rabbit anti-HSP70 (1:1,000, Abcam), rabbit anti-Alix (1:1,000, Abcam), and rabbit anti-HA (1:1,000, Abcam). Incubation was performed overnight at 4°C. Subsequently, the membranes were further incubated with horseradish enzyme-labeled goat anti-rabbit IgG or anti-mouse IgG (1:5,000, ZSGB-Bio). The final step involved imaging on an automated chemiluminescence image analysis system (4300, Tanon) digital image scanner using chemiluminescence.

### RNA isolation and qRT-PCR

Total RNA was acquired from engineered exosomes, serum, mouse hippocampus, and prefrontal cortex using TRIzol reagent (Invitrogen), and mRNA was reverse transcribed utilizing the qPCR kit (Genstar) and quantification through SYBR Green qPCR Master Mix (Genstar). The internal control typically employed was typically β-actin. Primers for all mRNA transcripts were synthesized by Sangon, and their sequences are detailed in Table S2.

### Immunofluorescence

Immunostaining and quantification procedures followed previously described methodologies [51–53]. Briefly, new neurons in the dentate gyrus (DG) region were identified using the anti-doublecortin (DCX) (1:600, Cell Signaling). Astrocytes were identified with the anti-GFAP (1:600, Cell Signaling). Microglia were labeled with anti-Iba1 (1:600, Cell Signaling) or anti-CD68 (1:600, Cell Signaling). Neurons were labeled with anti-MAP2 and anti-NeuN (1:600, Cell Signaling). Quantification was performed by capturing images with a microscope (Olympus BX51, Japan) equipped with a digital camera.

### Statistical analysis

All data in the study are presented as the mean ± SEM. The relative amount of qRT-PCR and fluorescence counting was analyzed using statistical methods such as 2-tailed Student’s *t* test or ANOVA. *P* ≤ 0.05 was regarded as statistically significant.

## Data Availability

The authors confirm that the data supporting the findings of this study are available within the article and its Supplementary Materials.
