# Supplementary material for: Targeted Delivery of Engineered RVG-BDNF-Exosomes: A Novel Neurobiological Approach for Ameliorating Depression and Regulating Neurogenesis
Source: Research (Wash D C). 2024 Jul 4;7:0402. doi: 10.34133/research.0402 (PMC12599878; doi:10.34133/research.0402)
Supplement: Supplementary 1 — Figs. S1 to S9 Tables S1 and S2 [file research.0402.f1.zip › Supplementary manuscript.docx]

**Targeted Delivery of Engineered RVG-BDNF-Exosomes: A Novel Neurobiological Approach for Ameliorating Depression and Regulating Neurogenesis**

Shaobo Liu^1#^, Lei Chen^1#^, Mei Guo^1^, Yongbiao Li^1^, Qingshan Liu^1*^, Yong Cheng^1*^

^1^ Key Laboratory of Ethnomedicine for Ministry of Education, Center on Translational Neuroscience, Minzu University of China, Beijing 100000, China.

*** Correspondence:**
Corresponding Author：Yong Cheng; yongcheng@muc.edu.cn

Supplementary Table 1 Demographic and clinical characteristics of HC, MDD and MDD-Treatment samples

| Characteristics | HC | MDD | MDD-Treatment |
| --- | --- | --- | --- |
| Demographic characteristics |  |  |  |
| Total, n | 20 | 20 | 20 |
| Age, median | 25 | 25.5 | 25 |
| Female, n (%) | 10(50) | 10(50) | 11(55) |
| Smoking history | No | No | No |
| BDNF, pg/ml, mean (SEM) | 469.17(146.14) | 209.56(104.66) | 539.97(219.53) |
| HAMD Score | 3(1.94) | 37.5(3.75) | 11(6.39) |

Supplementary Table 2

Sequence of the primers used in qRT-PCR.

| primers | Forward sequence 5’-3’ | Reverse sequence 5’-3’ |
| --- | --- | --- |
| BDNF | GGCTTGACATCATTGGCTGAC | CATTGGGCCGAACTTTCTGGT |
| TNF-α | CCCTCACACTCAGATCATCTTCT | GCTACGACGTGGGCTACAG |
| IL-6 | TAGTCCTTCCTACCCCAATTTCC | TTGGTCCTTAGCCACTCCTTC |
| IL-10 | GCTCTTACTGACTGGCATGAG | CGCAGCTCTAGGAGCATGTG |
| IL-4 | GGTCTCAACCCCCAGCTAGT | GCCGATGATCTCTCTCAAGTGAT |
| β-Actin | GGCTGTATTCCCCTCCATCG | CCAGTTGGTAACAATGCCATGT |

Supplementary Figure 1

Figure S1. Preparation and characterization of human serum exosomes (A) Size distribution and concentration of exosmes were analyzed by ZetaView. (B) Transmission electron microscopy images of exosmes. Scale bar, 100 nm. (C) Characteristic expression of lysates of cells in peripheral blood and their released exosomes as measured by western blot analysis. (D-F) (D: F _(2, 12)_ = 45.52, *P* < 0.0001; F: F _(2, 12)_ = 6.603, *P* = 0.0116) Expression level of BDNF was detected in lysates of cells in peripheral blood and their released exosomes by qRT-PCR (n = 5) and western blot analysis (n = 5). The data were normalized to β-Actin or CD63expression and expressed as mean ± SEM.**P* < 0.05, *****P* < 0.0001). Three or more groups were compared using one-way ANOVA followed by tukey's tukey's multiple comparison test.

Supplementary Figure 2

Figure S2 illustrates the distribution of normal exosomes (Normal-Exos) and RVG-Vector Exos in the DG area and prefrontal cortex region of normal mice 8 hours after intravenous injection. (A and E) Depicts the distribution of Normal-Exos in the DG region and prefrontal cortex region. (B and F) Depicts the distribution of RVG-Vector Exos in the DG region and prefrontal cortex region. (C and G) Presents the total number of exosomes distributed in the DG region and prefrontal cortex region by Normal-Exos and RVG-Vector Exos (C: *P* = 0.0330; G: *P* = 0.0002). (D and H) Shows the number of neuron-targeted exosomes distributed in the DG region and prefrontal cortex region by Normal-Exos and RVG-Vector Exos (D: *P* = 0.0470; H: *P* = 0.0002). n=3, Two groups were compared using t-test. **P* < 0.05, ***P* < 0.01, ****P* < 0.001 compared with the Normal-Exos group. Red dots represent Dil+ Exos, and greenish fluorescent light represents NeuN. Scale bar = 50 μm.

Supplementary Figure 3

Figure S3 illustrates the distribution of RVG-Exos in the DG region and prefrontal cortex region of normal mice (Normal-RVG-Exos) as well as LPS-induced model mice (LPS-RVG-Exos) 8 hours after intravenous injection. (A and E) Shows the distribution of Normal-RVG-Exos in the DG region and prefrontal cortex region. (B and F) Illustrates the distribution of LPS-RVG-Exos in the DG region and prefrontal cortex region. (C and G) Presents the total number of exosomes distributed in the DG region and prefrontal cortex region by Normal-RVG-Exos and LPS-RVG-Exos (C: *P* = 0.5177; G: *P* = 0.7937). (D and H) Depicts the number of neuron-targeted exosomes distributed in the DG region and prefrontal cortex region by Normal-RVG-Exos and LPS-RVG-Exos (D: *P* = 0.6964; H: *P* = 0.8418). n=3, Two groups were compared using t-test. Ns in comparison with the Normal-Exos group. Red dots represent Dil+ Exos, and greenish fluorescent light represents NeuN. Scale bar = 50 μm.

Supplementary Figure 4

Figure S4 After 12 hours of RVG-Exos incubation, the distribution in primary nervous system cells was observed, including A: neurons (MAP2), B: neurons (NeuN), C: astrocytes (GFAP) and D: microglia (IBA-1). Red dots, Dil + Exos; greenish fluorescent light, MAP2, NeuN, GFAP or IBA-1, Bar = 20 um.

Supplementary Figure 5

Figure S5 The distribution of RVG-Exos in A: neurons (MAP2) , B: astrocytes (GFAP) and C: microglia (IBA-1) 3 days after intravenous injection. Red dots, Dil + Exos; greenish fluorescent light, NeuN, GFAP or IBA-1, Bar = 20 um.

Supplementary Figure 6

Figure S6 RVG-BDNF-Exos improved CRS induced depression-like behavior in mice. (A-D) BDNF improved depression-like behavior in CRS mice as measured by the Open Field Test (A) (F _(3, 28)_ = 11.6, *P* < 0.0001), Forced Swimming Test (B) (F _(3, 28)_ = 10.91, *P* < 0.0001), Novelty-Suppressed Feeding Test (C) (F _(3, 28)_ = 56.08, *P* < 0.0001), and Tail Suspension Test (D) (F _(3, 28)_ = 11.88, *P* < 0.0001). n = 8 animals/group. Three or more groups were compared using one-way ANOVA followed by tukey's tukey's multiple comparison test. * *P* < 0.05, *** *P* < 0.001 and **** *P* < 0.0001 versus the CRS or RVG-Vector-Exos group.

Supplementary Figure 7

Figure S7 RVG-BDNF-Exos attenuates microglia activation in DG, CA1, CA2, CA3 and prefrontal cortex. Blue fluorescent light, DAPI; greenish fluorescent light, IBA-1; purple fluorescent light, CD68. Bar = 100 um.

Supplementary Figure 8

Figure S8. RVG-BDNF-Exos reduced the levels of blood cytokines and spleen weight of depressive mice

(A-E) The qRT-PCR experiments examined the effects of RVG-BDNF-Exos on TNF-α (A) (F _(3, 16)_ = 61.19, *P* < 0.0001), IL-6 (B) (F _(3, 16)_ = 100.5, *P* < 0.0001), IL-10 (C) (F _(3, 13)_ = 12.97, *P* = 0.0003), IL-4 (D) (F _(3, 16)_ = 11.94, *P* = 0.0002) and spleen (E) (F _(3, 20)_ = 15.81, *P* < 0.0001). n= 4-6. Three or more groups were compared using one-way ANOVA followed by tukey's tukey's multiple comparison test. **P*<0.05, ***P* < 0.01, ****P* < 0.001, *****P* < 0.0001 in comparison with the model or RVG-Vector-Exos group.

Supplementary Figure 9

Figure S9 RVG-BDNF-Exos is taken up in smaller amounts by microglia and astrocytes. A: microglia; B: astrocyte. Blue fluorescent light, DAPI; greenish fluorescent light, IBA-1 or GFAP. Bar = 100 um.
